# Changes in the Physiological Parameters of *SbPIP1*-Transformed Wheat Plants under Salt Stress

**DOI:** 10.1155/2015/384356

**Published:** 2015-10-01

**Authors:** G. H. Yu, X. Zhang, H. X. Ma

**Affiliations:** Provincial Key Laboratory of Agrobiology, Jiangsu Academy of Agricultural Sciences, Nanjing 210014, China

## Abstract

The SbPIP1 gene is a new member of the plasma membrane major intrinsic gene family cloned from the euhalophyte *Salicornia bigelovii* Torr. In order to understand the physiological responses in plants that are mediated by the SbPIP1 gene, SbPIP1-overexpressing wheat lines and WT plants of the wheat cv. Ningmai 13 were treated with salt stress. Several physiological parameters, such as the proline content, the malondialdehyde (MDA) content, and the content of soluble sugars and proteins, were compared between SbPIP1-transformed lines and WT plants under normal growth or salt stress conditions. The results indicate that overexpression of the SbPIP1 gene can increase the accumulation of the osmolyte proline, decrease the MDA content, and enhance the soluble sugar biosynthesis in the early period but has no influence on the regulation of soluble protein biosynthesis in wheat. The results suggest that SbPIP1 contributes to salt tolerance by facilitating the accumulation of the osmolyte proline, increasing the antioxidant response, and increasing the biosynthesis of soluble sugar in the early period. These results indicate SbPIP1 plays an important role in the salt stress response. Overexpression of SbPIP1 might be used to improve the salt tolerance of important crop plants.

## 1. Introduction

Water uptake is a vital function of terrestrial plant roots for survival. The growth of terrestrial plants will be seriously inhibited when water uptake through the roots is reduced by water-related stress such as drought and salt stress. Aquaporins are involved in plant response to water-related stress [1–5]. Aquaporins are membrane intrinsic proteins with a molecular mass of approximately 30 kDa. Aquaporins belong to a major intrinsic protein (MIP) super family with six transmembrane helices that facilitate the permeation of water through biomembranes [6]. Plant aquaporins are mainly divided into two types according to their subcellular localization: plasma membrane intrinsic proteins (PIPs) and tonoplast membrane intrinsic proteins (TIPs) [7].

Overexpression of the* Arabidopsis* plasma membrane aquaporin (PIP1b) in transgenic tobacco plants increased the plant growth rate, transpiration rate, stomatal density, and photosynthetic efficiency under favorable growth conditions, whereas PIP1b overexpression had no beneficial effect under salt stress and even a negative effect during drought stress, causing faster wilting [8]. Yu et al. showed that overexpression of BnPIP1 from* Brassica napus* in transgenic tobacco plants resulted in an increased tolerance to water stress [9]. The aquaporin RWC3 was upregulated during a water deficit in upland rice and RWC3-transformed lowland rice plants showed an improved water status during a water deficit [10]. Gao et al. reported that the overexpression of a putative aquaporin gene from wheat, TaNIP, enhanced salt tolerance in transgenic* Arabidopsis* [11]. Zhou et al. reported that constitutive overexpression of the soybean plasma membrane intrinsic protein GmPIP1;6 confers salt tolerance [12]. Xu et al. reported that overexpression of MaPIP1;1 from banana in transgenic* Arabidopsis* plants confers drought and salt stress tolerance by maintaining osmotic balance, improving ion distribution, and reducing membrane injury [13]. Overexpression of MusaPIP2;6 enhanced the salt tolerance in transgenic banana plants and MusaPIP2;6-overexpressing banana plants displayed better photosynthetic efficiency and lower membrane damage under salt stress conditions [14].

Despite the increase in the number of reports demonstrating the roles of aquaporins in plant response to environmental stresses, most studies have focused on the research of aquaporin function in glycophytes. Research on aquaporin function in halophytes is limited. Yamada et al. reported that the transcript accumulation of McMipA and McMipC (members of a family of MIP-related genes) was correlated with turgor recovery following salt-induced water stress in the ice plant (*Mesembryanthemum crystallinum*, moderate salt-tolerant) which is a halophyte; McMipA- and McMipB-encoded proteins expressed in* Xenopus* oocytes led to increased water permeability [15]. Decreases in the photosynthetic rate and stomatal conductance were less significant in McMIPB-overexpressing tobacco plants than in control plants when plants were grown under the soil water deficit condition [16].

*Salicornia bigelovii* Torr. is a euhalophyte that requires sodium (100 to 400 mM NaCl) for optimal growth.* S. bigelovii* can grow well with seawater irrigation, indicating that it has developed good molecular and physiological systems for adaptation to salt stress conditions. Therefore,* S. bigelovii* is a valuable model plant to characterize the genes responsible for water-related stress tolerance in plants. A new member of the plasma membrane major intrinsic gene family (*SbPIP1*) was cloned from* S. bigelovii* [17]. The* SbPIP1* gene was transformed into the wheat genome of cv. Ningmai 13 and the salt tolerance analysis of the transgenic lines during the germination period showed that the salt tolerance of the* SbPIP1-*transformed lines was better than that of Ningmai 13 [18]. We hypothesize that* SbPIP1* plays an important role under salt stress conditions.

The purpose of this study was to reveal the mechanism of enhanced salt tolerance mediated by the* SbPIP1* gene in plants.* SbPIP1*-overexpressing wheat lines and WT plants (Ningmai 13) were subjected to salt stress. Various physiological parameters, such as the proline content, the MDA content, and the contents of soluble sugars and soluble proteins, were compared between* SbPIP1*-transformed lines and WT plants under normal growth or salt stress conditions. Our results provide mechanistic details of salt tolerance conferred by the* SbPIP1* gene in correlation with the physiological changes observed. This study is the first report to explain the mechanism of enhanced salt tolerance in wheat, which is an important worldwide crop, mediated by an aquaporin gene from a halophyte.

## 2. Materials and Methods

### 2.1. Plant Material and Salt Stress Condition

Seeds from four transformed wheat lines (variety Ningmai 13) harboring the* SbPIP1* gene [18] were sown and raised inside a net house. Seedlings of the four transformed lines were tested using Basta spray for the selection of true transgenic plants and were raised for two months in the net house, after which they were transferred from pots to glass tubes (containing 40 mL of Hoagland's nutrient solution) and cultured in an incubator for 7 days under controlled conditions of 70%–75% relative humidity, 16 h of light, and an average temperature of 25°C. Seedlings of the Ningmai 13 variety (WT, wild type) were raised and cultured as the transgenic lines at the same time. In the salt stress treatment, Hoagland's nutrient solution was replaced with Hoagland's nutrient solution containing 250 mM NaCl in the glass tubes. The leaves from the WT and transgenic wheat plants were collected at 0, 1, 2, and 3 days and used for the experiments. In each treatment, the leaves of 3 seedlings were mixed and used for analysis. All the tests were carried out in triplicate.

### 2.2. Assay of Proline Content

The proline content in wheat leaves was estimated according to Shah and Dubey [19]. Fresh leaf samples (0.2 g) were homogenized in 5 mL of 3% aqueous sulphosalicylic acid and then centrifuged at 12,000 rpm for 10 min. Acid ninhydrin (2 mL) and glacial acetic acid (2 mL) were added to 2 mL of supernatant. The mixture was boiled in a water bath at 100°C for 1 h and then extracted with 4 mL of toluene. The absorbance of chromophore was measured at 520 nm using toluene as a blank. L-Proline (Merck) was used to construct the standard curve. The proline concentration (*μ*g/g FW) was calculated as follows: proline content (*μ*g) × extraction solution volume (mL)/sample volume (mL) × fresh leaf sample weight (g).

### 2.3. Measurement of MDA Content

Fresh leaf samples (0.2 g) were homogenized in 10 mL of 10% trichloroacetic acid and centrifuged at 12000 rpm for 10 min. The supernatant (2 mL) was added to 2 mL of 0.6% thiobarbituric acid (TBA) and incubated in a water bath at 100°C for 15 min. The mixture was centrifuged at 12,000 rpm for 10 min after it had cooled. The supernatant was measured at 532, 600, and 450 nm. The MDA content was calculated as follows: MDA content (uM) = 6.45 (OD532−OD600) − 0.56 OD 450.

### 2.4. Measurement of Soluble Protein Concentration

A standard curve for protein concentration was determined using the absorbance values of known concentrations of bovine serum albumin (0, 20, 40, 60, 80, and 100 *μ*g) at 595 nm. Fresh leaf samples (0.2 g) were homogenized in 4.0 mL phosphate buffer (0.01 M, pH = 7.0), and centrifuged at 12,000 rpm for 10 min. 4 mL Coomassie brilliant blue G250 solution was added to the supernatant (2 mL). The light absorbance of the mixture was measured at 595 nm, and the protein concentration was calculated using the standard curve. The soluble protein concentration (mg/g FW) was calculated as follows: protein content (mg) × extraction solution volume (mL)/sample volume (mL) × fresh leaf sample weight (g).

### 2.5. Soluble Sugar Content Assay

A standard curve for sugar mass was created by using absorbance values of known concentrations of glucose (0, 20, 40, 60, 80, and 100 *μ*g) at 620 nm. Fresh leaf samples (0.2 g) were homogenized in 4 mL of water, boiled in a water bath at 100°C for 30 min, and centrifuged at 12,000 rpm for 10 min. The supernatant was diluted with water to 10 mL, and a 0.2 mL aliquot of anthrone solution (0.5 g anthrone dissolved in 500 mL of 80% sulfuric acid solution) was added and then incubated at 100°C for 10 min. The absorbance values were measured at 620 nm and the sugar content was calculated using the standard curve. The soluble sugar concentration (%) was calculated as follows: sugar content (*μ*g) × extraction solution volume (mL) × dilute fold × 100/sample volume (mL) × fresh leaf sample weight (g) × 10^6^.

## 3. Results

### 3.1. Assessment of Salt Tolerance

**Figure 1:**
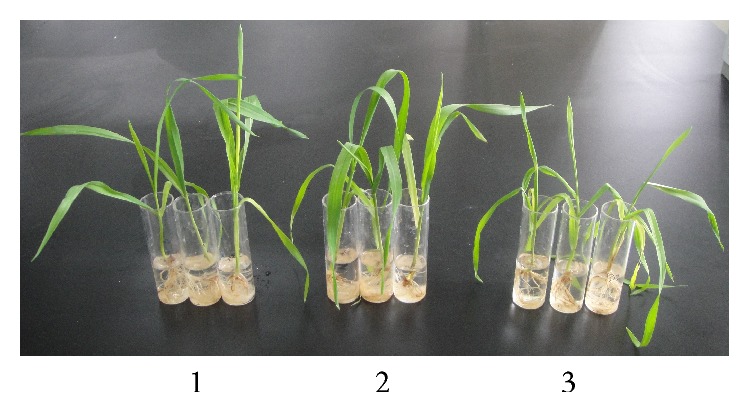
The influence of salt stress on transgenic lines and WT plants. 1: WT plants without stress; 2: transgenic lines D1 after 3 days of salt stress; 3: WT plants after 3 days of salt stress. WT represents the untransformed wheat variety Ningmai 13.

The growth of the transgenic lines and WT plants were compared following exposure to 250 mM NaCl stress. The growth of* SbPIP1*-transformed lines was generally not affected by salt treatment. There were almost no differences between the growth of transgenic plants after 3 days of salt stress and the growth of WT plants without stress. However, the growth of the WT plants was remarkably affected by salt treatment. The leaves of WT plants had clearly wilted after 3 days of salt stress (Figure 1). These results indicate that* SbPIP1* transgenic lines retain a salt-tolerant phenotype.

### 3.2. Accumulation of Proline Content under Salt Stress

**Figure 2:**
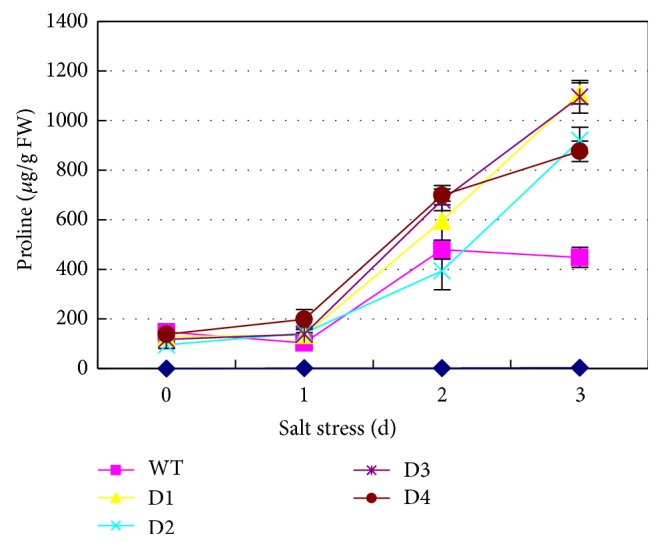
Proline content in WT plants and* SbPIP1* gene transgenic lines. D1, D2, D3, and D4 represent the transgenic lines D1, D2, D3, and D4, respectively; WT represents the untransformed wheat variety Ningmai 13.

The proline content in all transgenic wheat lines increased with an increase in the duration of the salt stress (Figure 2). The proline content in WT plants increased during the first two days of salt stress and then dropped slightly by the 3rd day. Overall, the proline contents in the plants overexpressing the* SbPIP1* gene were significantly (*P* < 0.01) higher than those in the WT plants after 3 days of stress treatment (Table 1).

### 3.3. MDA Content Assay

**Figure 3:**
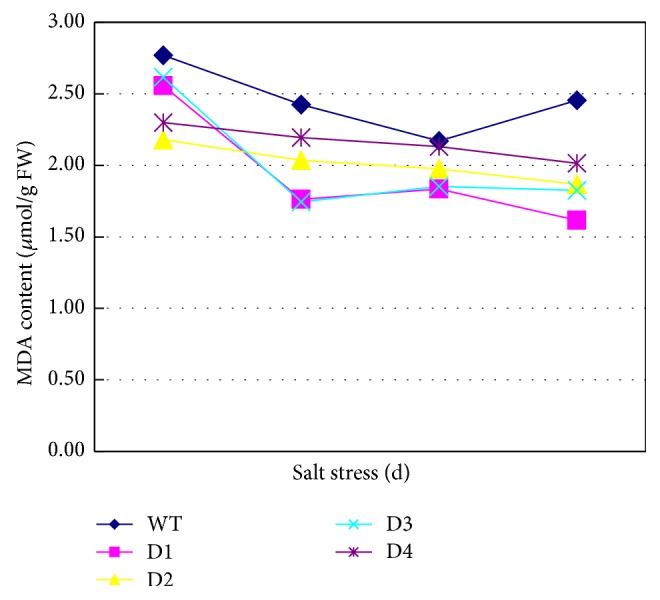
MDA content in the WT plants and* SbPIP1* gene transgenic lines. D1, D2, D3, and D4 represent the transgenic lines D1, D2, D3, and D4, respectively; WT represents the untransformed wheat variety Ningmai 13.

The MDA content in the transgenic lines and WT plants was analyzed (Figure 3). During salt stress treatment with NaCl, the MDA contents changed in all of the treatment groups, that is, the transgenic lines and the WT plants. The MDA content in the WT plants decreased during the first two days and then increased on the 3rd day. The MDA contents in all of the transgenic lines except the transgenic line D3 exhibited a declining trend during the three days of salt stress; the MDA content in the transgenic line D3 decreased after 1 day of stress treatment and then increased slightly over the subsequent days of treatment. Overall, the MDA contents in plants overexpressing the* SbPIP1* gene were significantly (*P* < 0.05) lower than those in the WT plants after 3 days of stress treatment, indicating that lipid peroxidation was lower in the* SbPIP1*-transformed lines than in the WT plants. These results suggest that the* SbPIP1* gene might contribute to decreased lipid peroxidation in wheat.

### 3.4. Soluble Protein Content Assay

**Figure 4:**
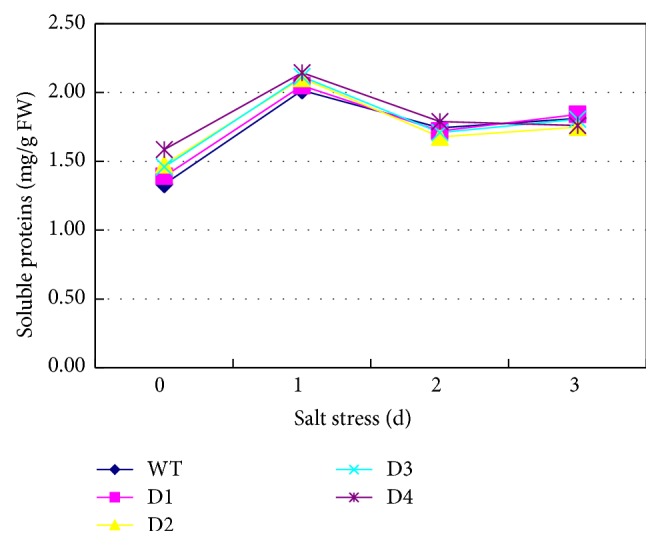
Soluble protein content in the WT plants and* SbPIP1* gene transgenic lines. D1, D2, D3, and D4 represent the transgenic lines D1, D2, D3, and D4, respectively; WT represents the untransformed wheat variety Ningmai 13.

The soluble protein content was measured in the* SbPIP1*-transformed lines and the WT plants (Figure 4). An almost identical change trend was exhibited in all the treated groups, that is, the transgenic wheat lines and the WT plants, during the salt stress treatment. The soluble protein content increased approximately 0.5-fold after 1 day of stress treatment, followed by a slight decrease after 2 days of stress treatment, and then remained fairly constant after exposure to salt stress for 3 days.

### 3.5. Soluble Sugar Content Assay

**Figure 5:**
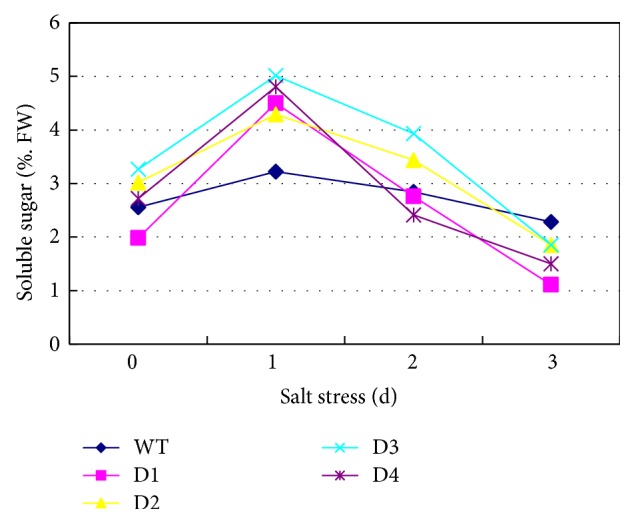
Soluble sugar content in the WT plants and* SbPIP1* gene transgenic lines. D1, D2, D3, and D4 represent the transgenic lines D1, D2, D3, and D4, respectively; WT represents the untransformed wheat variety Ningmai 13.

The soluble sugar content was analyzed in both the transgenic wheat plants and the WT plants (Figure 5). Under salt stress conditions (250 mM NaCl treatment), the soluble sugar contents changed in all treatment groups; however, there were distinct differences in the soluble sugar content between the* SbPIP1*-transformed plants and the WT plants. The soluble sugar content increased 0.5-fold in WT plants after 1 day of stress treatment and then decreased slowly over the subsequent days of treatment. In the* SbPIP1*-transformed plants, the soluble sugar content increased greatly (approximately 1-fold) after 1 day of stress treatment and then decreased dramatically over the subsequent days of treatment. As shown in Figure 5, the soluble sugar levels in all transgenic plant groups dropped to the same level as the WT plants after 3 days of salt stress treatment. Although the soluble sugar content differed among the three transgenic plant lines, a similar change trend was exhibited in all transgenic plant lines during the salt stress treatment. Overall, the soluble sugar content changes observed in the* SbPIP1*-transformed plants differed from those observed in the WT plants, suggesting that the* SbPIP1* gene plays a role in the regulation of soluble sugar biosynthesis in wheat.

## 4. Discussion

It is known that proline has various functions in plants under stress conditions. This amino acid can serve as an eliminator of free radicals, a mediator of osmotic adjustment, a buffer of redox potential, a stabilizer of inserted subcellular structures, and an important component of cell wall proteins [20, 21]. The proline content accumulates considerably in plants subjected to salt and drought stress due to its increased synthesis or decreased degradation [22–24]. Therefore, the proline content in plants under salt stress can be a vital criterion for evaluating salt tolerance. Gao et al. reported that TaNIP-overexpressing* Arabidopsis* accumulated more proline than the wild-type plants [11]. In addition, MaPIP1;1-overexpressing transgenic* Arabidopsis* plants maintained higher levels of proline compared with WT plants subjected to a similar drought treatment [13]. In the current study, a significant increase in proline accumulation was observed in transgenic wheat plants after salt stress treatment, and this was correlated with enhanced tolerance to salt stress.

Key enzymes in the lipid metabolic pathways in plants are affected by salt stress [25]. Lipid peroxidation is considered the most damaging process in living organisms. Since MDA is one of the end-products of lipid peroxidation in biomembranes, the MDA content is usually used to represent the level of lipid peroxidation and membrane injury. The MDA content is an important criterion in evaluating the stress tolerance of plants under stress conditions. MaPIP1;1-overexpressing* Arabidopsis* exhibited a reduced MDA content under salt or drought conditions [13]. In addition, TaAQP7- (a PIP2 subgroup aquaporin gene) overexpressing tobacco plants and TaAQP8- (a PIP1 subgroup aquaporin gene) overexpressing tobacco plants had lower levels of MDA than the WT plants under salt stress [26, 27]. The MDA levels were lower in transgenic banana plants overexpressing MusaPIP2;6 than in the untransformed plants under salt stress condition [14]. In this study, the MDA levels in plants overexpressing the* SbPIP1* gene were significantly (*P* < 0.05) lower than those in the WT plants after 3 days of stress treatment. This result showed that overexpression of the* SbPIP1* gene may significantly reduce lipid peroxidation in transgenic wheat plants.

The soluble protein content in different plant species changes in different ways under salt stress. In salt-tolerant plant species such as rice, barley, and sunflower [28], the soluble protein content increases under salt stress. In mulberry cultivars, the soluble protein content increased at a low salinity level but decreased at a high salinity level [29]. The overexpression of GmCLC1 (a vacuolar Cl^−^ transporter protein gene) in poplar led to an increase in the soluble protein content during salt stress [30]. The soluble protein content in transgenic tobacco (overexpressing a bZIP transcription factor gene from* Medicago sativa* L.) increased compared with nontransgenic tobacco under salt or drought stress [31]. In the current study, the soluble protein levels in the transgenic lines and WT wheat plants exhibited an almost identical change trend. We hypothesize that overexpression of the* SbPIP1* gene has no influence on the regulation of soluble protein biosynthesis in wheat.

Under abiotic stress conditions, plant cells accumulate different types of osmolytes to adjust the intracellular osmotic potential and avoid cell injury. Among many different kinds of osmolytes, soluble sugars are the major types of osmolytes. The major role played by soluble sugars in stress mitigation involves osmoprotection, carbon storage, and scavenging of reactive oxygen species [32]. The overexpression of GhAnn1 (a cotton annexin gene) in transgenic cotton plants conferred enhanced salt tolerance with higher levels of soluble sugars compared with the wild-type plants [33]. The overexpression of OsDREB2A in soybean enhanced salt tolerance, which was accompanied with an accumulation of soluble sugars [23]. In the current study, we examined the soluble sugar content in transgenic wheat plants overexpressing the* SbPIP1* gene and found that the soluble sugar levels were significantly (*P* < 0.05) higher than those in the WT plants after 1 day of salt stress treatment (Figure 5), after which the soluble sugar content decreased more dramatically than that in the WT plants. Based on these data, we hypothesize that the* SbPIP1* gene may contribute to the synthesis of soluble sugars during the early period (1 day) of salt stress treatment.

The overexpression of several PIP1 genes such as BnPIP1, NtAQP1, TaAQP8, OsPIP1;1, MusaPIP1;2, and MaPIP1;1 enhanced the hydraulic conductance and tolerance of transgenic plants in response to water stress [9, 13, 27, 34–36]. Aharon et al. (2003) reported that AtPIP1b overexpression in transgenic tobacco plants had no beneficial effect under salt stress and even a negative effect during drought stress, causing faster wilting [8]. Zhou et al. (2014) reported that transgenic soybean overexpressing GmPIP1;6 exhibited higher growth and greater yield under salt treatment compared with the WT plants [12]. However, the mechanism of how some PIP1 genes can increase the tolerance to water stress is largely unknown. Maintaining osmotic balance, improving ion distribution, reducing membrane injury, and enhancing the activities of antioxidants were mentioned with respect to the overexpression of the PIP1 genes [13, 26, 27]. In this study, the overexpression of* SbPIP1* led to increases in the accumulation of proline and the synthesis of soluble sugars and reduced lipid peroxidation. From the results in this study, we confirmed that* SbPIP1* plays an important role in the salt stress response in halophytes and glycophytes. The overexpression of* SbPIP1* in plants might be used to improve the salt tolerance of important crop plants.

## Figures and Tables

**Table 1:** Proline content in the WT plants and *SbPIP1* gene transgenic lines after 3 days of 250 mM NaCl treatment.

Lines	Proline content (*μ*g/g)	5% level	1% level
WT	448.333	c	C
D1	1109.39	a	A
D2	920.067	b	B
D3	1095.407	a	A
D4	876.037	b	B
